# Prevalence of hepatitis B virus infection among health care workers in a tertiary hospital in Tanzania

**DOI:** 10.1186/s12879-015-1129-z

**Published:** 2015-09-23

**Authors:** A. Mueller, L. Stoetter, S. Kalluvya, A. Stich, C. Majinge, B. Weissbrich, C. Kasang

**Affiliations:** Bugando Medical Centre, P.O. Box 1370, Mwanza, United Republic of Tanzania; CUHAS, P.O. Box 1370, Mwanza, United Republic of Tanzania; Institute of Virology and Immunobiology, University of Wuerzburg, Versbacher Strasse 7, 97078 Wuerzburg, Germany; Medical Mission Institute, Salvatorstrasse 7, 97067 Wuerzburg, Germany; Department of Tropical Medicine, Medical Mission Hospital, Salvatorstrasse 7, 97074 Wuerzburg, Germany

**Keywords:** Hepatitis B virus, Hepatitis C virus, Health care workers, Point-of-care test, Tanzania

## Abstract

**Background:**

Sub-Saharan Africa has a high prevalence of hepatitis B virus (HBV) infections. Health care workers (HCWs) are at high risk of contracting HBV infection through their occupation. Vaccination of HCWs against HBV is standard practice in many countries, but is often not implemented in resource-poor settings. We aimed with this cross-sectional study to determine HBV prevalence, HCW vaccination status, and the risk factors for HCWs contracting HBV infection in Tanzania.

**Methods:**

We enrolled 600 HCWs from a tertiary Tanzanian hospital. Their demographics, medical histories, HBV vaccination details and risk factors for contracting blood-borne infections were collected using a standardized questionnaire. Serum samples were tested for HBV and hepatitis C virus (HCV) markers by ELISA techniques, PCR and an anti-HBs rapid test. HCWs were divided in two subgroups: those at risk of contracting HBV (rHCW 79.2 %) via exposure to potentially infectious materials, and those considered not at risk of contracting HBV (nrHCW, 20.8 %).

**Results:**

The overall prevalence of chronic HBV infection (HBsAg+, anti-HBc+, anti-HBs-) was 7.0 % (42/598). Chronic HBV infection was found in 7.4 % of rHCW versus 5.6 % of nrHCW (*p*-value = 0.484). HCWs susceptible to HBV (HBsAg-, anti-HBc-, anti-HBs-) comprised 31.3 %. HBV immunity achieved either by healed HBV infection (HBsAg-, anti-HBc+, anti-HBs+) or by vaccination (HBsAg-, anti-HBc-, anti-HBs+) comprised 36.5 % and 20.2 %, respectively. 4.8 % of participants had indeterminate results (HBsAg-, anti-HBc+, anti-HBc-IgM-, anti-HBs-). Only 77.1 % of HCWs who received a full vaccination course had an anti-HBs titer >10 ml/U. An anti-HBs point-of-care test was 80.7 % sensitive and 96.9 % specific. There was a significantly higher risk for contracting HBV (anti-HBc+) among those HCW at occupational risk (rHCW) of older age (odds ratios (OR) in rHCW 3.297, *p* < 0.0001 vs. nrHCW 1.385, *p* = 0.606) and among those HCW being employed more than 11 years (OR 2.51, *p* < 0.0001***). HCV prevalence was low (HCV antibodies 1.2 % and HCV-RNA 0.3 %).

**Conclusions:**

Chronic HBV infection is common among Tanzanian HCWs. One third of HCWs were susceptible to HBV infection, highlighting the need for vaccination. Due to high prevalence of naturally acquired immunity against HBV pre-testing might be a useful tool to identify susceptible individuals.

## Background

Worldwide, more than 2 billion people are infected with hepatitis B virus (HBV). Of these, 240 million are chronic carriers of HBV and are at risk of death from acute fulminant liver disease, liver cirrhosis or hepatocellular carcinoma (HCC). The World Health Organization (WHO) has stated that the prevalence of hepatitis B is highest in sub-Saharan Africa and East Asia, and they estimate that between 5–10 % of the adult population are chronically infected [1]. Currently, several drugs like tenofovir and entecavir have been approved in industrialized countries for the therapy of chronic HBV infection according to established guidelines from professional medical organizations [2]. Antiviral treatment of chronic hepatitis B infection significantly delays the progression of cirrhosis, reduces the incidence of HCC and improves long-term survival [3]. However, in many resource-constrained settings, including Tanzania, implementation of this treatment has not occurred.

The lack of treatment opportunities in resource-constrained settings makes prevention of HBV infection crucial. In countries with high HBV prevalence, most HBV transmission occurs already during childhood; however, a significant proportion of people remains susceptible to HBV and is, therefore, at risk of contracting the virus during their adult age [4, 5].

Hepatitis B is an important occupational hazard for health care workers (HCW) [1]. In some studies, HCWs have been shown to have an up to four-fold increased risk of acquiring HBV infection [4, 5]. The main risk factor to contract HBV infection for HCWs is direct contact with infectious material, especially HBV-infected blood or via a needle stick injury with HBV-contaminated body fluids [6]. In particular, recapping of hollow-bore needles appears to increase the risk of needle stick injuries [7]. Other studies have reported a lack of awareness of HBV among HCWs; consequently, proper precautions (e.g., use of disposable gloves) against blood-borne infections are lacking in these workers [8]. This observation is consistent with other studies demonstrating that untrained individuals are more likely to be exposed to HBV infection [5, 9].

Preventive vaccination against hepatitis B for hospital staff is standard in many countries, but is still not implemented in many resources-poor settings [10, 11]. There have been reports of weak immune responses to HBV vaccination caused by, for example, diabetes or a current viral infection [12–14]. Therefore the WHO recommends to monitor immune responses to the vaccine in addition to compulsory vaccination of HCWs [15].

In Tanzania, the prevalence of acute or chronic HBV infection among blood donors or adults in Dar es Salaam, Tanzania, was found to be 8.8 % [16, 17]. and 6 %, respectively. Furthermore, a study conducted on women in a rural area of north-eastern Tanzania found that previous contact with HBV was common, with 74 % of them being HBV-positive as defined by anti-hepatitis B core antibody (anti-HBc) detection [18]. The high prevalence of hepatitis B in Tanzania poses not only a risk to HCWs, but also to non-immune patients who risk being infected by a HCW with chronic hepatitis B infection. This is especially applicable to situations involving invasive medical procedures like surgery [19]. To date, there have not been any reports in the scientific literature on HBV infection and HBV immune status in Tanzanian HCWs. Studies on the prevalence of chronic hepatitis B and C in HCWs from other sub-Saharan African countries are scanty. Kateera et al. reported a prevalence of 2.9 % chronic hepatitis B infection, indicated by positive HBsAg, and 1.3 % anti-HCV-positivity among tertiary hospital employees in Rwanda [20]. Ziraba et al. found chronic hepatitis B infection in 8.1 % of the HCWs in a central teaching hospital in Uganda [5]. In Nigerian healthcare workers a HBsAG-prevalence of 13 % was reported by Ola et al. [21]. Apparently there are considerable differences in the rate of chronic HBV-infection among HCWs in sub-Saharan Africa.

With the assumption that the prevalence of hepatitis B in HCWs will be at least as high as in the general population, about 30 % of HCWs remain susceptible to hepatitis B. Non-immune HCWs have a high risk of contracting an infection at the place of work and would benefit from vaccination against HBV therefore.

There are no national recommendations or vaccination program against HBV for HCWs in Tanzania. However, in 2002 [22] Tanzania implemented the WHO policy of general HBV vaccination for children as part of the extended program on immunization (known as EPI). According to notified data, a vaccination coverage of 91 % of 1-year-old children was achieved in 2013 [23]. A limited national health budget dictates that the most cost-effective strategy should be found allowing to implement preventive HBV vaccination for hospital personnel at risk. Such a strategy should consider the high rate of naturally acquired HBV infection among adults in endemic countries [6]. To distinguish immune HCWs from those who are HBV susceptible, laboratory tests are essential. Since conventional HBV serology is costly and often not available in resource-poor settings, cheaper alternatives need to be found. An expanding range of point-of-care tests for infectious diseases including HBV offer logistical advantages at low cost [24] and with a sensitivity and specificity comparable to standard methods.

An accurate point-of-care-test for hepatitis B surface antibodies (anti-HBs) could identify those HCWs who are already immune and do not need to be vaccinated therefore. Taking the comparatively high cost of the HBV vaccine itself and the logistical costs for the three required vaccinations into account, this approach should be more cost-effective than an untargeted vaccination program. Here, we aimed to provide data on the prevalence of acquired immunity against HBV, HCV and chronic infection with these viruses in HCWs in a tertiary hospital in Tanzania. The secondary objectives were the risk of infection for HCWs at work over time, their vaccination status, and the performance of a commercial anti-HBs point-of-care-test for use prior to vaccination.

## Methods

### Study design and setting

This hospital-based study was conducted in 2012 at the Bugando Medical Centre (BMC) in Mwanza, Tanzania and used a cross-sectional design. BMC is a referral and teaching hospital with a 1000 bed capacity serving a population of about 13 million people in north-western Tanzania. The hospital engages about 1400 employees in different professions.

### Participants and samples

The study enrolled 600 HCWs aged ≥16 years. In 598 of the enrolled participants a complete data set was available for analysis. The study was announced within the hospital by poster, blackboard, presentations in the lecture hall and oral announcement. The participation was voluntary and 600 participants were enrolled consecutively until the desired number of participants was met. The questionnaire was prepared taking into account the most common professions within the hospital setting. The questionnaire did not include questions concerning sexual behavior as this was considered culturally inappropriate in the study setting.

Eligible were employees of the hospital of Tanzanian origin. Medical students and nursing students were included as they have regular duties in patient care. Expatriates were excluded from the study.

After giving informed consent, a standardized questionnaire was used to collect information on demographics, medical history, profession and the HBV immunization status of all the participants. The language of the interview was English or Swahili according to the preference of the participant. For one sub-analysis, the HCWs were divided into two subgroups by their profession. Subgroup 1.A: At occupational risk HCWs (rHCWs) comprising medical doctors, medical students, nurses, nursing students, laboratory personnel and cleaning personnel who are frequently exposed to infectious materials such as used needles. Subgroup 2.A: Not-at-occupational risk HCWs (nrHCWs) comprising administration staff, technical service staff and allied health sciences staff. An additional sub-analysis was conducted on a subgroup classified by exposure to needlestick injury, surgery, blood transfusion, intravenous (i.v.) drug use, intramuscular (i.m.)/i.v. injection; this subgroup was compared to the non-exposed study participants, regardless of their profession.

From each participant, 14 ml of whole blood was collected into two serum sampling tubes for serological analysis. The samples were centrifuged for 3 min at 1650 g and the serum was stored in cryovials at −20 °C. For shipment the samples were packed in a special insulation box to maintain a temperature of −20 °C.

### Ethical issues

The study was approved by the CUHAS/BMC Research Committee (BREC) joint ethics review board (Research Clearance Certificate: BREC/001/06/2012). All participants signed an informed consent form that covered sample analysis for HBV and HCV. Testing for HIV was explicitly excluded as it was assumed to interfere with the willingness to participate in the study. Each participant received a study number. No personal data was recorded at any time. The individual test results were made available to the hospital in a sealed envelope labelled with the specific study number. Each participant was able to collect his letter anonymously, containing the laboratory results and their interpretation. In case of findings indicating a chronic Hepatitis B the participant was advised to seek medical care for further evaluation.

### Benefit for the study participant

Although currently no antiviral treatment is licensed for chronic hepatitis B infection in Tanzania knowledge of the hepatitis B status is of benefit for the patient, as he is able to undergo further monitoring and evaluation. In addition, it allows family members and potential sexual partners to be vaccinated. In case of women of child-bearing age prevention of mother-to-child transmission is possible. Those participants with significant anti-HBs titers were reassured of their Hepatitis B immunity, those without previous infection were advised to be vaccinated against HBV. These aspects were carefully considered by the ethical board of the hospital.

### HBV and HCV analyses

All serological analyses were performed in Germany after transfer of the samples from Tanzania. Serum samples were available for analysis in 598 of 600 participants. All serum samples were tested for HBs-antigen, anti-HBc antibodies and quantitative anti-HBs-antibodies. In addition, HBs-antigen-positive samples were tested for anti-HBc IgM antibodies, HBe-antigen and anti-HBe antibodies. The following test kits were used for HBV serology: Enzygnost HBsAg 6.0 (Siemens Healthcare, Marburg, Germany), Enzygnost anti-HBc monoclonal (Siemens), Architect Anti-HBs (Abbott, Wiesbaden, Germany), and Architect Anti-HBc-IgM. All 598 serum samples were screened for hepatitis C antibodies. Anti-HCV-positive samples were analyzed by PCR for HCV RNA. HCV antibodies were identified using the Architect Anti-HCV assay (Abbott). In addition, 592 serum samples were tested with further anti-HBs antibody test (Hepatitis B antibody test no. BSB3120001, SureScreen Diagnostics, Derby, Great Britain), which was an easy-to-use point-of-care test in cassette format. The test utilizes a double antigen sandwich system and detects anti-HBs levels as low as 10 U/mL in whole blood, serum or plasma.

### Statistical analysis

Data were entered into a Microsoft Excel database. Statistical analyses were performed using IBM SPSS Version 18 software and GraphPad Prism version 6.01 for Windows, GraphPad Software, La Jolla California USA, http://www.graphpad.com/scientific-software/prism/. Chi-square and Fisher exact tests were used to assess associations between categorical variables.

## Results

### Patient characteristics

Four age groups were defined: 16–30 years (198/33.1 %), 31–40 years (178/29.8 %), 41–50 years (120/20.1 %) and 51–65 years (102/17.1 %). Among all participants, 360 (60.2 %) were female. Out of the study participants 473 (79.2 %) were stratified as rHCWs and 125 (20.8 %) as nrHCWs according to the questionnaire.

### HBV and HCV prevalence

**Fig. 1:**
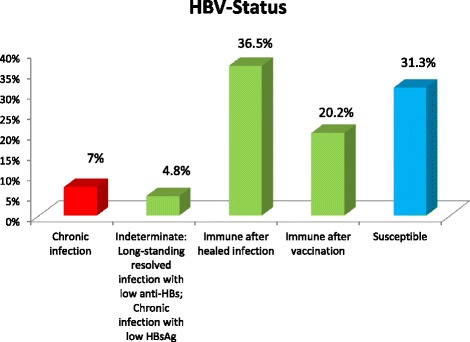
HBV- status in HCWs in Tanzania. Prevalence of chronic HBV infection (HBsAg+, anti-HBc+, anti-HBs-), HBV immunity achieved by healed HBV infection (HBsAg-, anti-HBc+, anti-HBs+) or by vaccination (HBsAg-, anti-HBc-, anti-HBs+), indeterminate result (HBsAg-, anti-HBc+, anti-HBs-) and HBV susceptibility (HBsAg-, anti-HBc-, anti-HBs-) in Tanzanian HCWs in a tertiary hospital as determined by HBV serology. HBV: hepatitis B virus; HCWs: health care workers

The overall prevalence of chronic HBV infection (HBsAg+, anti-HBc+, anti-HBs-) was 7.0 % (42/598). Chronic HBV infection was found in 7.4 % of rHCWs versus 5.6 % of nrHCWs, with no statistical significance difference between these groups (*p*-value = 0.484). Those still susceptible to HBV infection comprised 31.3 % of the HCWs (HBsAg-, anti-HBc-, anti-HBs-). HBV immunity achieved in HCWs either by healed HBV infection (HBsAg-, anti-HBc+, anti-HBs+) or by vaccination (HBsAg-, anti-HBc-, anti-HBs+) was found in 36.5 % and in 20.2 % respectively. A serum marker constellation considered as healing HBV infection (HBsAg+, anti-HBc+, anti-HBs+) was found in one participant (0.2 %). We found that 29 participants (4.8 %) had indeterminate results (HBsAg-, anti-HBc+, anti-HBc-IgM-, anti-HBs-) with isolated anti-HBc positivity caused by long-standing resolved infections with low anti-HBs titers or current infections with low HBsAg titers (Table 1 and Fig. 1).

**Table 1:** HBV infection status of HCWs from a Tanzanian tertiary hospital

HBV serological markers			Interpretation	HCW (*n* = 598)	rHCW (*n* = 473)	nrHCW (*n* = 125)	*p*-value
HBsAg	Anti-HBc	Anti-HBs (U/mL)			subgroup	subgroup	
+	+	<10	Chronic infection	42/7.0 %	35/7.4 %	7/5.6 %	0.484
-	-	<10	Susceptible	187/31.3 %	144/30.4 %	43/34.4 %	0.498
-	+	>10	Immune after healed infection	218/36.5 %	171/36.2 %	47/37.6 %	0.765
-	-	>10	Immune after vaccination	121/20.2 %	99/20.9 %	22/34.4 %	0.432
+	+	>10	Healing infection	1/0.2 %	1/0.2 %	---	--
-	+	<10	Indeterminate: Long-standing resolved infection with low anti-HBs; Current infection with low HBsAg	29/4.8 %	23/4.9 %	6/4.8 %	0.977

*HBV* hepatitis B virus, *HCW* health care worker

Subgroups: rHCWs who were frequently exposed to infectious materials and therefore at risk of contracting HBV infection, and nrHCWs who were considered not to be at risk of contracting HBV infection

HCV prevalence was low, with HCV antibodies of 1.2 % and HCV RNA of 0.3 %. There was no statistically significant difference between the rHCW and nrHCW groups (p-value HCV-Antibodies: 0.668, HCV-RNA: 0.309), and no co-infections of HBV and HCV. Due to the low prevalence of HCV-infection no further statistical analyses were performed.

### HBV vaccinations in HCWs

Out of the 598 HCWs, 380 (63.5 %) stated in their questionnaires that they had been vaccinated against HBV. Also, 292 (48.8 %) of them had received three doses of the vaccine in the last 10 years, while 60 (10 %) had received two vaccinations, and 27 (4.5 %) only one vaccination. One participant was vaccinated more than 10 years ago. In the group vaccinated three times within the last 10 years, anti-HBs positive results were found in 225 (77.1 %) of them. No laboratory confirmation of successful vaccination was done in the past.

### Point-of-care rapid testing

Sera analyzed with the point-of-care SureScreen Rapid Test Cassette were positive in 272 of 337 Architect anti-HBs positive samples (sensitivity 80.7 %). The cut off limit of Architect anti-HBs is 10 IU/L. Eight samples of 255 (3.1 %) Architect anti-HBs negative tests were positive by SureScreen Rapid Test (specificity 96.9 %). Six tests were not done because of a lack of material (Table 2).

**Table 2:** Sensitivity and Specificity of Surescreen anti-HBs Rapid test compared to Architect anti-HBs

	Architect anti-HBs negative <10 IU/L	Architect anti-HBs positive >10 IU/L
Rapid test anti-HBs negative	247	65
Rapid test anti-HBs positive	8	272
Total	255	337

Specificity: anti-HBs10 = 247/255 = 96.9 %

Sensitivity: anti-HBs10 = 272/337 = 80.7 %

### HBV risk factors

Some risk factors were found to be significantly associated with chronic hepatitis B infection (HBsAg+) and the risk to contract HBV-infection (anti-HBc+) at a 5 % level of significance (Table 3). There was no significant difference for contracting HBV (anti-HBc+) between males and females (OR females 0.8897; *p* = 0.5044), but females had a statistically significant lower risk to develop chronic infection (HBsAg+) (OR females 0.4484; *p* = 0.0146).

**Table 3:** Risk factors for contracting hepatitis B virus and current HBV infection

Variables	Current HBV Infection (HBsAg +)		Contracting HBV (Anti HBc+)	
	Odds ratio	*P*–value	Odds ratio	*P*–value
Gender (Ref = Male)				
Female	0.45 [0.24–0.84]	0.0146*	0.89 [0.64–1.24]	0.5044
Age (Ref =16–30)				
31–40	0.91 [0.43–1.90]	0.8526	1.43 [0.95–2.16]	0.0939
41–50	0.76 [0.32–1.82]	0.6687	1.75 [1.06–2.88]	0.0304*
51–65	0.43 [0.14–1.33]	0.1574	2.77 [1.69–4.53]	<0.0001***
Work duration (Ref = 0–5)				
6–10	1.59 [0.74–3.42]	0.2892	1.45 [0.92–2.28]	0.1286
>11	0.74 [0.35–1.58]	0.4650	2.51 [1.74–3.63]	<0.0001***
Profession (Ref = Administration)				
Medical Doctors (Surgeons, Physicians, Students)	3.69 [0.81–16.86]	0.0923	1.56 [0.87–2.82]	0.1767
Nursing staff	2.41 [0.54–10.77]	0.3816	1.00 [0.58–1.70]	1
Laboratory personnel	1.29 [0.06–28.71]	1	1.54 [0.40–5.96]	0.7370
Allied Sciences	1.34 [0.12–15.44]	1	0.64 [0.26–1.62]	0.3674
Technical Services	5.15 [0.89–29.87]	0.0667	1.18 [0.50–2.78]	0.8275
Cleaning Staff	2.70 [0.54–13.40]	0.3042	1.52 [0.81–2.84]	0.2057
Risk factors (Ref = Yes)				
Blood transfusion	0.44 [0.10–1.88]	0.4156	1.02 [0.59–1.76]	1
Operation	0.97 [0.48–1.99]	1	1.08 [0.75–1.55]	0.7103
i.m./i.v.drug administration	1.47 [0.44–4.90]	0.7883	1.50 [0.86–2.61]	0.1677
Needle stick injury	0.96 [0.50–1.84]	1	1.12 [0.80–1.56]	0.5504

**Fig. 2:**
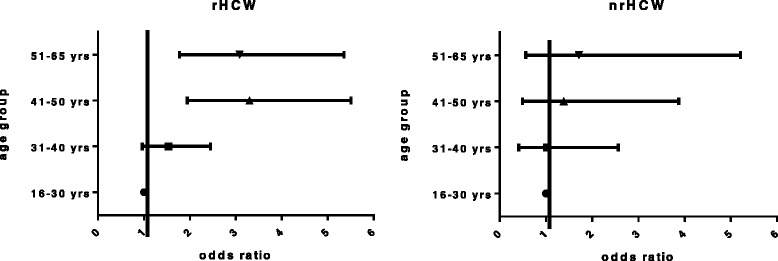
Risk of HCWs contracting HBV by age. Risk of contracting HBV (based on anti-HBc-positivity tests) by age group in health care workers at occupational risk (rHCW) or those not at occupational risk (nrHCW). Data are shown as a risk factor odds ratio table. HBV: hepatitis B virus

**Fig. 3:**
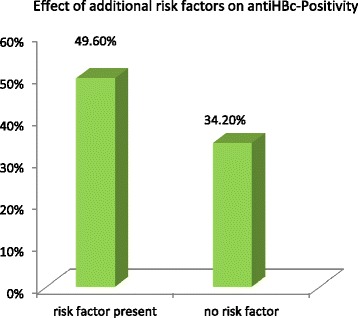
Exposure to HBV in Tanzanian HCWs with and without additional risk factors. Anti-HBc positivity (contact with HBV) in HCWs with additional risk factors (49.6 %) versus professionals without additional risk factors (34.2 %; *p* = 0.065 Chi square Test).

A significantly higher risk for contracting HBV was identified by estimating the anti-HBc odds ratios in the different age groups. The results showed a statistically significant correlation between age of the HCW and acquisition of markers of HBV. The odds ratio in 51–65 year-old group of all HCW compared to 16–30 year-olds was 2.766 (*p* = <0.0001). This result is consistent with the fact, that the odds ratio in participants with a working duration of more than 11 years compared to those with a working duration of less than 5 years was 2.511 (*p* = <0.0001).When separated into the two subgroups of HCW at occupational risk and those not at occupational risk the odds ratio for contracting HBV (anti-HBc+) in the 51–65 year-old group compared to 16–30 year-olds in the rHCW group was 3.297 (*p* < 0.0001) versus nrHCW 1.385(*p* = 0.606) (Fig. 2). Overall, we found an increase of anti-HBc positivity in HCWs with risk factors (49.6 %) versus individuals without risk factors (34.2 %; *p* = 0.065, Chi square test) but there was no statistically significant difference (Fig. 3).

## Discussion

This study revealed a high burden of HBV infection in HCWs in Tanzania. The overall prevalence of chronic HBV infection (HBsAg positivity) among HCWs in a tertiary hospital in the northern part of Tanzania was 7.0 %. Immunity (anti-HBs positivity) was present in 56.9 % of workers but 31.3 % of them were still susceptible to HBV infection. Immunity against HBV was achieved via a healed infection in 36.5 % of the HCWs or via vaccination in 20.2 % of this group. The findings from a similar study conducted among HCWs in Uganda, which borders the northern part of Tanzania, found a comparable prevalence of current HBV infection (8.1 %) but a higher prevalence of HBV susceptibility (48.9 %) [5].

The proportion of HCWs who achieved immunity due to healed infection with HBV in this study (36.5 %) was higher than that of a different study on outpatients (25.3 %) [25] in Tanzania, suggesting that HCWs have a high risk of becoming infected with HBV through their occupation. Additionally, we found a greater than 3-fold higher risk for HCWs to acquire an infection with HBV during their life, especially when they are on occupational risk e.g. handling blood and needles (odds ratio 3.297, *p* < 0.0001).

The study found a significantly higher rate of HBV exposure in older HCW than in younger ones. This could be explained by various reasons. One explanation could be that there is a more or less constant risk of exposure during life time and therefore the Hepatitis B prevalence increases with age. We cannot rule out, that the risk of transmission might have changed over time due to increased awareness and precautions like wearing of gloves and use of safety needles.

On the other hand the finding, that long occupational exposure in healthcare services increases the risk of acquiring HBV infection, is consistent with other studies [5, 26, 27]. Up to now, Tanzania has not performed a controlled nationwide vaccination campaign against hepatitis B in high risk groups like HCW’s [28]. In the past, the special setting of this tertiary health care facility made it possible to start decentralized HBV vaccination programs in HCWs supported by donations. Hence, the proportion of HCWs (20.2 %) who received full vaccinations is higher than in other studies [5, 25]. But the vaccination programs conducted thus far have been of doubtful efficacy. Indeed, 63.5 % of the HCWs stated in their questionnaires that they were vaccinated against HBV, but only 48.8 % of the vaccinated had received three shots within the last 10 years. Within this group, immunity to HBV could be demonstrated in only 77.1 % by detection of anti-HBs antibodies. This leaves 22.9 % of the remaining HCWs in doubtful protection despite feeling well protected. Their doubtful status of immunity remains unnoticed without laboratory confirmation of a sufficient antibody titer against the virus.

Even though in resource-limited settings most HBV infections occur perinatal or secondarily during early childhood, there is still a high risk of HCWs becoming infected via occupational exposure to the virus. In HBV-endemic developing countries where people have high rates of natural immunity, the provision of universal HBV vaccination for HCWs is often discussed because of questionable cost effectiveness of this preventive strategy [6, 29]. It can be assumed, that an approach avoiding unnecessary vaccinations could reduce the costs of a vaccination program. Hence, introduction of a point-of-care test for anti-HBs in a pre-vaccination screening phase for HCWs could be cost-effective. Use of such a test could reduce the number of HCWs requiring vaccination, avoid unnecessary vaccinations and save vaccine for individuals already being immune. The SureScreen anti-HBsAb-Test used in this study had a sensitivity of 80.7 % and a specificity of 96.9 %. Using this rapid diagnostic test, 46.8 % of the participants were identified, for various reasons, as being immune to HBV; hence, these HCWs will require no further vaccinations against HBV. For the above reasons, pretesting of the HBV status of HCWs could be worthwhile before implementing a nationwide vaccination program for HCWs, providing a test of high sensitivity and specificity is available. The test used in this study has the limitation, that the detected specificity is low. Aside from the existing policy in Tanzania on injection safety in health care settings, use of auto-disposable syringes and better safety awareness concerning HBV as an infectious agent are crucial to the prevention of blood-borne infections.

### Hepatitis C

Data concerning HCV infection in Sub-Saharan Africa are scanty. Therefore we screened the samples also for HCV antibodies. We found an HCV-antibody prevalence of 1.2 % with only 2 chronic HCV infections demonstrated by RNA detection.

Compared to our results in the Democratic Republic of Congo 13.7 % of the samples were seropositive for HCV but only 3.7 % were viraemic [29]. Other African studies from Nigeria and Angola showed rates of HCV seroprevalence (anti-HCV+) of 12.8 % and 8.1 % [30, 31].

### Limitations of the study

Voluntary participation in a study has always limitations resulting from a recruitment bias. Our study might not reflect a representative sample of the hospital staff. All information concerning previous vaccinations was obtained by questionnaire without validation by vaccination records. Concerning the efficacy of Hepatitis B vaccination a recall bias of the participants needs to be considered concerning the number and type of vaccinations.

## Conclusions

We found that the prevalence of chronic HBV infection and the risk of occupational exposure to HBV among HCWs were both high in a tertiary hospital in northern Tanzania. One third of HCWs were found to be susceptible to HBV infection. This finding highlights the need for HBV vaccination among HCWs. Currently, protection of HCWs against HBV in this tertiary hospital is beyond the desired level because they have not been vaccinated, the vaccine has failed to induce protection, or they have not received all three doses. Laboratory confirmation of immunization success was not conducted. Taking into account the high prevalence of naturally acquired immunity anti-HBs testing could avoid unnecessary immunizations. For this purpose highly sensitive and specific point-of-care tests would be needed. The cost effectiveness of such an approach should be analyzed in further studies. The Tanzanian government recognizes the importance of HBV vaccination for children; the next step could be to consider adults at risk, such as HCWs, for a targeted vaccination campaign.
